# Clinical and Haemodynamic Evaluation of a Novel Physician Modified Inner Branch Iliac Branch Device for East Asians

**DOI:** 10.1016/j.ejvsvf.2026.01.005

**Published:** 2026-01-30

**Authors:** Mingwei Wu, Li Zhang, Shichao Liang, Wei Guo, Jiang Xiong

**Affiliations:** a Department of Vascular and Endovascular Surgery, Chinese PLA General Hospital, Beijing, China; b Department of Vascular and Endovascular Surgery, Dazhou Central Hospital, Dazhou, China; c School of Life Science, Beijing Institute of Technology, Beijing, China

**Keywords:** Abdominal aortic aneurysm, Common iliac artery aneurysm, Computational fluid dynamics, Endovascular aortic repair, Iliac branch device

## Abstract

**Introduction:**

The anatomic suitability of the iliac branch device remains limited, particularly in East Asians, in whom the common iliac arteries (CIAs) are notably short. This study aimed to evaluate the safety and haemodynamic effects of the physician modified inner branch iliac branch device (PM-IIBD) via clinical outcomes and computational fluid dynamics (CFD) analysis.

**Method:**

In this observational case series study, clinical safety was evaluated in ten patients treated with the PM-IIBD for internal iliac artery preservation. CFD analyses were performed before surgery and at 12 month follow up to compare haemodynamic changes in four CIA regions. CFD parameters included velocity, pressure, time averaged wall shear stress (TAWSS), relative residence time, oscillatory shear index (OSI), energy loss, and flow distribution ratios.

**Results:**

Technical success was 100%, with no peri-operative or follow up adverse events. CFD revealed minimal changes in pressure and velocity after PM-IIBD implantation, with only a slight post-operative decrease in external iliac artery average velocity (0.51 ± 0.12 m/s *vs*. 0.45 ± 0.16 m/s, *p* = .039). High oscillatory shear index areas in the CIA region significantly decreased after surgery (0.22 ± 0.14 *vs*. 0.11 ± 0.07, *p* = .036). In the PM-IIBD region, both the average TAWSS (0.40 ± 0.45 *vs*. 0.72 ± 0.22, *p* = .015) and maximum TAWSS increased statistically significantly (4.47 ± 4.16 *vs*. 8.44 ± 6.52, *p* < .001). The PM-IIBD inner branch region showed an increase in high TAWSS areas (0.11 ± 0.39 *vs*. 0.20 ± 1.44, *p* = .002). Energy loss decreased significantly after surgery (3.82 ± 2.13 *vs*. 3.11 ± 1.76, *p* = .013), with no significant changes in the relative residence time and flow distribution ratio.

**Conclusion:**

Preliminary clinical and CFD analyses have demonstrated the efficacy and haemodynamic stability of the PM-IIBD. By reducing spatial demands on the CIA, the PM-IIBD expands anatomic suitability, offering a feasible solution for internal iliac artery preservation in East Asians.

## Highlights

• Anatomic suitability of the Iliac Branch Device (IBD) is limited in East Asians.
• Physician Modified Inner Branch IBD (PM-IIBD) minimizes CIA spatial demands.
• Clinical and computational fluid dynamics validate PM-IIBD safety and efficacy.
• PM-IIBD expands IBD anatomic suitability given the anatomic constraints in East Asians.

## INTRODUCTION

Endovascular aortic repair (EVAR) offers a less invasive alternative to open surgery for abdominal aortic aneurysms (AAAs), reducing operative mortality and morbidity rates in anatomically suitable patients.^1^ However, the presence of concomitant common iliac artery aneurysms (CIAAs), found in 15–45.5% of AAA cases, presents significant challenges to EVAR.^2^^,^^3^ A critical factor for long term success in EVAR is the establishment of a sufficient distal landing zone in a healthy segment of the common iliac artery (CIA), which helps to prevent complications such as stent migration and type Ib endoleaks (T1bELs).^4^ To achieve this, endografts are often extended to the external iliac artery (EIA), which may require either embolisation or coverage of the internal iliac artery (IIA).^5^ However, sacrificing the IIA is associated with serious complications, including buttock claudication (28–42%), erectile dysfunction (17–24%), and risks of colonic or spinal cord ischaemia.^6^^,^^7^ The 2024 Clinical Practice Guidelines of the European Society for Vascular Surgery emphasise the preservation of at least one IIA during EVAR whenever feasible.^8^ Although iliac branch devices (IBDs) offer superior outcomes for IIA preservation, their applicability is limited by anatomic constraints, particularly in East Asian patients with narrower and shorter CIAs.^9^, ^10^, ^11^, ^12^ To address this challenge, a novel physician modified inner branch iliac branch device (PM-IIBD) that minimises spatial requirements was developed. This study aimed to assess the clinical safety and haemodynamic effects of the PM-IIBD via clinical outcomes and computational fluid dynamics (CFD) analysis.

## STUDY DESIGN

This observational case series study adhered to the reporting guidelines of the Strengthening the Reporting of Observational Studies in Epidemiology Statement.^13^ From February 2023 to May 2024, ten patients were enrolled from two centres after institutional review board approval (IRB S2023-597-01) and written informed consent.

## METHOD

### Patient selection

Inclusion criteria were as follows: (1) men with an AAA ≥55 mm or women with an AAA ≥50 mm combined with CIAA or isolated CIAA or IIAA with a maximum diameter ≥30 mm and (2) availability of computed tomography angiography (CTA) both before surgery and at 12 month follow up. Exclusion criteria were as follows: (1) age <18 years and (2) pseudo- or infected aneurysms.

### Data collection and definitions

**Figure 1:**
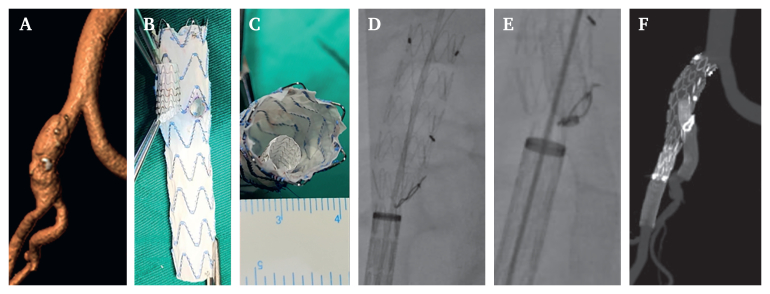
Physician modified inner branch iliac branch device (PM-IIBD) implantation process: (A) pre-operative computed tomography angiography showing the narrow inner lumen common iliac artery aneurysm; (B, C) PM-IIBD configuration; (D) partial release of the PM-IIBD with the inner branch opening aligned to the internal iliac artery; € reconstruction of the internal iliac artery via the inner branch of the inner branch iliac branch device; (H) post-operative computed tomography angiography at 12 month follow up.

Data on patient demographics (age, sex, body mass index, and comorbid chronic conditions) and pre-operative 1 mm slice thoraco-abdominopelvic CTA were obtained from the hospital's electronic medical record system (Fig. 1A). At 12 month follow up, a ≥5 mm reduction in aneurysm sac diameter was defined as sac shrinkage, an increase or reduction <5 mm as sac stability, and a ≥5 mm increase as sac dilation.^14^

### Study outcomes

The primary safety endpoint was freedom from major adverse events (death, myocardial infarction, renal or hepatic failure, respiratory failure, spinal cord ischaemia amputation, or puncture site complications) within 30 days after the operation. The primary efficacy endpoint was immediate post-operative technical success, with midterm outcomes assessed at the 12 month follow up, specifically absence of T1ELs and T3ELs, IIA branch stent patency, and freedom from re-intervention.

### Physician modified inner branch iliac branch device design

The PM-IIBD design and surgical procedure has been reported previously.^15^ The PM-IIBD was fashioned from a 16 × 13 × 95 mm mm Endurant II limb (Medtronic, Minneapolis, MN, USA) and a 7 × 150 mm inner branch cut from a Viabahn Endoprosthesis (WL Gore & Associates, Flagstaff, AZ, USA). The limb from the 18 F delivery system was released on the table and fenestrated with a cautery pen (Xinhai Hexing Technology, Dalian, China) and reinforced with 0.018 inch coil ring (Cook Medical LLC, Bloomington, IN, USA). The inner branch, featuring a 45° bevel and a diameter half that of the main PM-IIBD stent, was sutured proximally and distally with 5.0 polypropylene, ensuring parallel alignment (Fig. 1B and C). Subsequently, a puncture needle was employed to establish an orifice in the proximal sheath of the delivery system, enabling the V-18 pre-loaded guidewire (0.018 inch, Boston Scientific, Natick, MA, USA) to traverse through the inner branch and emerge from the proximal limb. Next, the inner branch iliac branch device (IIBD) was compressed using sutures and inserted into the 20 F Medtronic delivery system.

### Surgical procedure

Under general anaesthesia, both femoral arteries were cannulated. The pre-loaded V-18 guidewire from the inner branch was established as a through and through guidewire between the two femoral arteries. The PM-IIBD was delivered to the CIA bifurcation and partially released, with the fenestration aligned to the IIA origin (Fig. 1D). A 4 F catheter advanced via the through and through guidewire into the inner branch, enabling the release of the IIA branch stent (Fig. 1E). Finally, the PM-IIBD was fully deployed (Fig. 1F). Post-operative digital subtraction angiography revealed that the IIBD was well positioned, without T1ELs or T3ELs, stent migration, or kinking. The EIA and IIA remained patent.

### Image segmentation and 3D reconstruction

Image segmentation and 3D vessel configuration reconstruction were conducted using the automatic image processing module developed by the team, with the segmentation time of 14.2 ± 2 seconds for each case.^16^

### Computational fluid dynamics analysis of inner branch iliac branch device implantation

**Figure 2:**
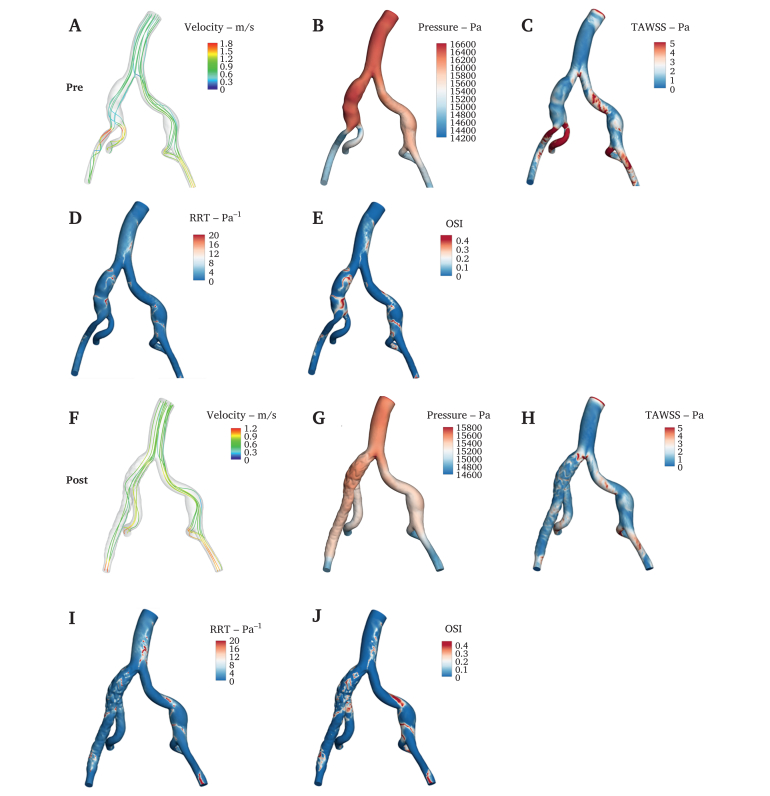
(A–E) Computational fluid dynamics analysis of aorto-iliac flow velocity, pressure, time averaged wall shear stress (TAWSS), relative residence time (RRT), and oscillatory shear index (OSI) in an isolated narrow lumen common iliac artery aneurysm before physician modified inner branch iliac branch device implantation; (F–J) corresponding computational fluid dynamics analysis 12 months after implantation of the physician modified inner branch iliac branch device, which is composed of an inner branch and limb.

The models were defined by sectioning perpendicular to the local vessel centreline at the inlet (infrarenal aorta) and outlets (distal EIA and IIA). Haemodynamic computations were conducted on all pre-operative and 12 month follow up models of ten patients. To conduct finite volume analyses, the aorta model was imported into ICEM (Ansys Inc., Canonsburg, PA, USA) for meshing, with tetrahedral elements in the vascular lumen and prismatic cells applied to the boundary layer. A finite volume solver (CFD-ACE 18.0, ESI Group, Paris, France) was used to solve the transport equations of time dependent flow. Pressure velocity coupling was handled using the SIMPLEC type pressure correction method. The boundary conditions were based on flow velocities and volumes data in the aorta, EIA, and IIA, from the measured pre- and post-operative ultrasound. The blood was modelled as a Newtonian fluid with a density of 1 044 kg/m^3^ and dynamic viscosity of 0.00365 Pa·s. The vessel wall was regarded as non-slip and rigid. The simulation was carried out for four cardiac cycles with 50 steps per cycle (each step 0.02 seconds, based on 60 beats/min heart rate, ensuring systolic and diastolic flow resolution), and the final cycle results were selected for post-processing to eliminate initial effects. Grid and time step independence tests were also necessary to ensure the base mesh resolution and time step settings were adequate. The CFD results were analysed using Tecplot 360 (Tecplot Inc, Bellevue, WA, USA). Haemodynamic parameters included velocity, pressure, time averaged wall shear stress (TAWSS), relative residence time (RRT), oscillatory shear index (OSI), energy loss (EL), and flow distribution ratios (FDRs) (Fig. 2). Moreover, TAWSS >4, RRT >20, and OSI >0.35 were regarded as high TAWSS, RRT, and OSI, respectively.^17^ The CFD analysis was also conducted on the high TAWSS, RRT, and OSI area changes after IIBD implantation. Visualisation results were also focused on four interested regions of the CIA (Fig. 4A), region 1 (the entire affected CIA), region 2 (the entire IIBD), region 3 (the IIBD main stent), and region 4 (the IIBD inner branch).

### Statistical analysis

Sample size was determined using a single variable non-inferiority test based on the binomial distribution. To assess interobserver variability of vessel measurements, the intraclass correlation coefficient (ICC) was calculated on the basis of independent measurements performed by two vascular surgeons (M.W. and L.Z.). Continuous variables were expressed as mean ± standard deviation, and categorical variables as frequencies and percentages. The ICC was used to assess interobserver variability. All analyses were conducted using SPSS (v26, IBM Corp., Armonk, NY, USA) and MATLAB (MathWorks, Natick, MA, USA).

## RESULTS

### Patient characteristics and data reliability

The study included ten patients, eight of whom were men. The mean age was 73.4 ± 7 years, and the mean body mass index was 23.5 ± 2 kg/m^2^. Regarding statistical power, for the sample size of 10, the calculated power was 0.904 (achieved α = .0282). Regarding measurement reliability, the ICC for measurements between the two surgeons was 0.913 (*p* < .001), indicating excellent agreement.

### Physician modified inner branch iliac branch device peri-operative outcomes

The technical success for PM-IIBD implantation was 100%. The average procedure time was 161.2 ± 30 minutes with a contrast dose of 172 ± 31 mL. No major adverse events, including death, myocardial infarction, renal failure, hepatic failure, respiratory failure, spinal cord ischaemia, limb amputation, infection, or puncture site complications, occurred during the 30 day post-operative period.

### Inner branch iliac branch device midterm follow up outcomes

All patients completed the 12 month post-operative follow up CTA examination. The results showed no IIBD or IIA branch stent migrations, no T1ELs or T3ELs, and no stenosis or occlusion in the EIA or IIA. None of the patients experienced buttock claudication, erectile dysfunction, colonic ischaemia, or spinal cord ischaemia during follow up. Additionally, there were no deaths or cardiovascular complications. In seven patients’ the sac shrank by ≥ 5 mm and in three it remained stable (Table 1).

**Table 1:** Physician modified inner branch iliac branch device (PM-IIBD) size and artery diameter changes before and after implantation.

Patient	PM-IIBD – mm	Inner branch – mm	Pre-max diameter – mm	Post–max diameter – mm	Diameter change – mm	EIA-IIA angle – °
1	16-16-120	8	32.6	27.4	−5.2	56.2
2	16-16-80	8	27.8	26.3	−1.5	31.3
3	16-13-95	8	34.5	14.3	−20.2	41.5
4	16-13-80	8	32.1	30.8	−1.3	39.4
5	16-13-90	8	56.3	48.6	−7.7	29.1
6	20-10-120	10	58.2	51.1	−7.1	41.4
7	20-16-95	10	45.0	38.4	−6.6	44.8
8	16-16-95	7	42.9	34.0	−8.9	47.1
9	14-10-170	7	39.8	40.2	+0.4	61.5
10	14-10-140	7	31.2	22.2	−9.0	54.3

PM-IIBD = physician modified inner branch iliac branch device; max = maximum; EIA = external iliac artery; IIA = internal iliac artery.

### Physician modified inner branch iliac branch device computational fluid dynamics analysis

#### Pressure and velocity changes after IIBD implantation

**Figure 3:**
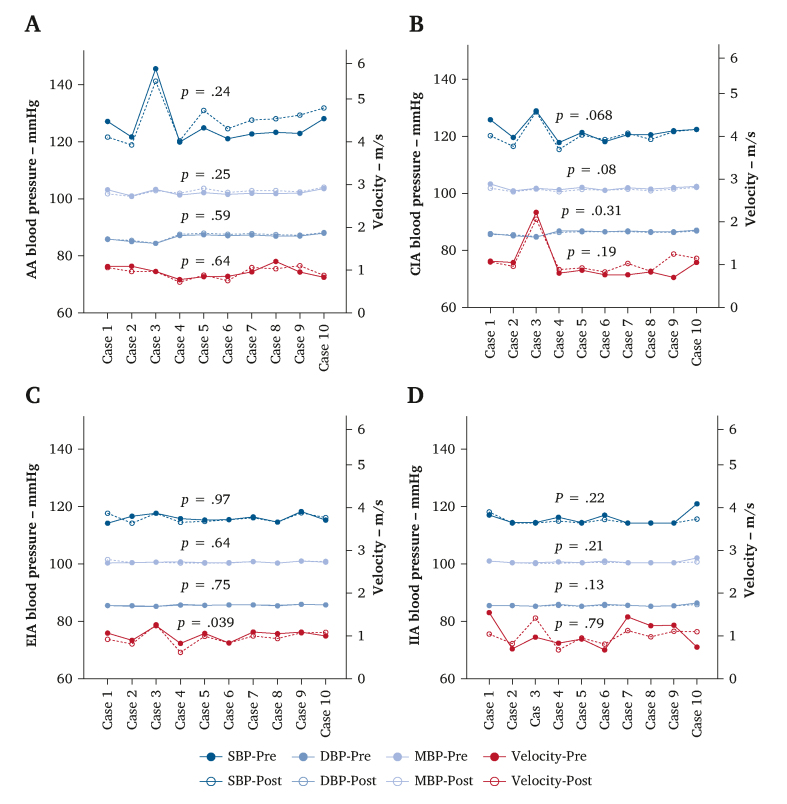
Pre- and post-operative changes in systolic blood pressure (SBP), diastolic blood pressure (DBP), mean blood pressure (MBP), and average velocity at various aorto-iliac regions by computational fluid dynamics simulations: (A) abdominal aorta (AA) region; (B) common iliac artery (CIA) region; (C) external iliac artery (EIA) region; (D) internal iliac artery (IIA) region.

CFD analysis of systolic pressure, diastolic pressure, mean pressure, and average velocity in the abdominal aorta, CIA, EIA, and IIA revealed minimal differences after implantation. The only significant change was a slight decrease in EIA average velocity after surgery (0.51 ± 0.1 m/s *vs.* 0.45 ± 0.1 m/s, *p* = .039) (Fig. 3).

#### Haemodynamic parameter changes after IIBD implantation

**Figure 4:**
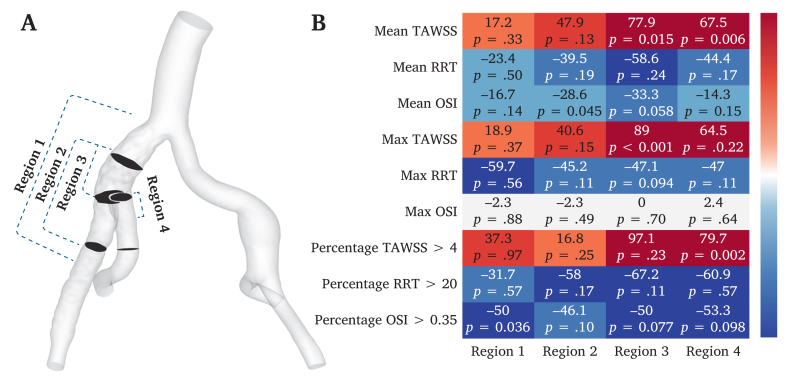
Four regions of the common iliac artery (CIA) analysed using computational fluid dynamics: region 1, entire CIA; region 2, entire physician modified inner branch iliac branch device (PM-IIBD); region 3, PM-IIBD main stent; and region 4, PM-IIBD inner branch. B Heatmap of haemodynamic parameter changes before and after PM-IIBD implantation in the four CIA regions. OSI = oscillatory shear index; RRT = relative residence time; TAWSS = time averaged wall shear stress.

CFD analysis of the CIA regions (regions 1–4) revealed significant changes in haemodynamic parameters (Supplementary Table S1). The following parameters were assessed: mean TAWSS, maximum TAWSS, mean RRT, maximum RRT, mean OSI, maximum OSI, and percentage areas for TAWSS >4, RRT >20, and OSI >0.35. Significant changes were observed in regions 1–4, whereas other parameters showed no significant differences (Fig. 4): (1) region 1 (the entire affected CIA), the percentage OSI >0.35 decreased after surgery (0.22 ± 0.14 *vs*. 0.11 ± 0.07, *p* = .036); (2) region 2 (the entire IIBD), the mean OSI decreased after surgery (0.07 ± 0.03 *vs*. 0.05 ± 0.02, *p* = .045); (3) region 3 (the IIBD main stent), both the mean TAWSS (0.40 ± 0.45 *vs*. 0.72 ± 0.22, *p* = .015) and maximum TAWSS (4.47 ± 4.16 *vs*. 8.44 ± 6.52, *p* < .001) increased; and (4) region 4 (the IIBD inner branch), the percentage TAWSS >4 increased after surgery (0.11 ± 0.39 *vs*. 0.20 ± 1.44, *p* = .002).

#### EL and FDR changes after IIBD implantation

Analysis of the CIA region's EL and FDR showed that EL decreased significantly after surgery (3.82 ± 2.13 *vs*. 3.11 ± 1.76, *p* = .013). No significant changes in FDR were observed for the EIA or IIA (EIA: 60% ± 14% vs. 56% ± 10%, p = .382; IIA: 40% ± 14% vs. 44% ± 10%, p = .382).

## DISCUSSION

In this study, the PM-IIBD was successfully implanted in ten CIA lesions, achieving a 100% technical success rate. No adverse events, including T1ELs, T3ELs, stent migration, stenosis, or occlusion, were observed during the peri-operative period or 12 month follow up. Seven patients exhibited aneurysm shrinkage, whereas three patients had stable aneurysm size. Importantly, there were no cases of buttock claudication, erectile dysfunction, colonic ischaemia, or spinal cord ischaemia, demonstrating the clinical midterm efficacy and safety of the PM-IIBD in CIA lesions. These favourable outcomes are particularly significant when considered against the landscape of current endovascular IIA preserving strategies, which, despite addressing the need for reconstruction, each possess inherent limitations. Although the bell bottom technique enables IIA preservation in lesions <25 mm, it carries high risks of aneurysmal dilatation (35%) and endoleaks (18%) with significant volume expansion reported for diameters ≥17 mm; meanwhile, parallel stent techniques (sandwich and crossover techniques) are associated with a relatively high T1EL rate due to gutter.^18^, ^19^, ^20^, ^21^ the IBD technique remains the most well established off the shelf solution for IIA preservation during EVAR, with superior early to midterm outcomes (97.3% technical success, 97.6% 30 day patency, and 94.3% follow up patency).^9^^,^^22^ Although IBDs offer superior outcomes, their application is restricted. Previous studies indicate that suitability rates can be as low as 9–28.8% in East Asian cohorts.^10^, ^11^, ^12^ A large scale anatomic study by Wu *et al.*^3^, involving 1 144 East Asian patients with AAA, found that only 18.9%, 21.8%, 11.9%, and 22.6% met the anatomic criteria for Cook ZBIS, Gore IBE, E-Liac IBD, and G-Iliac IBD, respectively. The primary anatomic limitation, insufficient CIA bifurcation diameter, affected 66.31% of cases. Lowering the CIA bifurcation threshold to 16 mm, 14 mm, or 12 mm could increase IBD anatomic eligibility by 19.4%, 39.1%, and 54.4%, respectively.^3^ Even in real world East Asian patient cohorts, the anatomic suitability is only approximately 50%.^23^ These findings emphasise the need to adjust IBD designs for reduced CIA diameter requirements and broader clinical-anatomic applicability. The advantages of the IIBD are as follows. Firstly, it has reduced CIA spatial requirements compared with existing IBDs. Secondly, it provides a longer proximal landing zone for the IIA branch stent (15 mm *vs.* 10 mm in IBDs), lowering the risk of stent migration. Thirdly, it facilitates the use of more flexible self expanding stents in tortuous IIA anatomies, whereas existing IBDs often require balloon expandable stents owing to limited landing zone length.

CFD has been used extensively to evaluate novel CIA stent grafts.^17^ By analysing the pre- and post-implantation changes in CFD parameters, it can be used to predict their effects on local blood flow stability and long term patency.^24^, ^25^, ^26^ In this study, the CIA with PM-IIBD implantation was subdivided into four regions for CFD analysis. This approach offers two advantages: firstly, validating the consistency and accuracy of CFD analysis across different regions; and secondly, identifying which part exhibits suboptimal blood flow patterns after PM-IIBD implantation to guide PM-IIBD optimisation. In this research, CFD analysis of ten patients with implanted PM-IIBD showed minimal changes in pressure and velocities in the abdominal aorta, CIA, EIA, and IIA. The only significant change was a slight post-operative decrease in EIA flow velocity. This decrease may be attributed to the inner branch structure of the PM-IIBD, which partially obstructed blood flow away from the EIA. However, FDR analysis showed no significant changes in EIA or IIA flow, suggesting that the slight decrease in EIA velocity did not significantly impact EIA flow. Additionally, CFD simulations revealed significant improvements in haemodynamics within the PM-IIBD region. The mean and maximum TAWSS increased after surgery. Higher TAWSS is associated with the maintenance of endothelial function, promoting vascular homeostasis and smooth blood flow, suggesting that PM-IIBD implantation leads to improved blood flow patterns within the PM-IIBD region.^27^ A decrease in the percentage of OSI >0.35 in the CIA region and PM-IIBD region was observed. The OSI is an indicator of flow oscillation, and a reduction suggests that the blood flow became more stable after PM-IIBD implantation.^28^^,^^29^ Furthermore, the reduction in RRT, a parameter reflecting the residence time of blood particles in the aorta, lowers the risk of atherosclerosis and thrombosis.^30^^,^^31^ In this study, no significant differences were observed in RRT, indicating that the PM-IIBD did not increase the risk of thrombosis within CIA region. Finally, EL, a parameter that quantifies mechanical ELs from shunting and turbulence during blood flow and is typically elevated in arterial stenosis, aneurysms, or bifurcations, was also assessed.^32^ In this study, the EL of ten patients was all reduced after PM-IIBD deployment, indicating improved blood flow efficiency. Overall, clinical midterm outcomes and CFD analysis support the PM-IIBD's effectiveness in improving haemodynamics and maintaining stable blood flow in CIA lesions. Thus, the PM-IIBD shows promise in overcoming the anatomic spatial limitations of outer branch IBDs, making it a valuable option for IIA preservation, particularly in East Asian populations. It is important to note that this study represents a preliminary feasibility exploration of the PM-IIBD technique. As a physician modified solution, it currently requires on table fabrication, which increases surgical complexity and limits widespread applicability. Consequently, the ultimate objective is to lay the groundwork for a commercial off the shelf IIBD. Regarding anatomic suitability, current clinical experience suggests specific boundaries. Although no complications were observed in the cohort with EIA-IIA angles ranging from 29.1° to 61.5°, the device may not be suitable for extreme iliac tortuosity (e.g., >90°), which could impede cannulation, result in kinking, or lead to detachment of the inner branch suture. Additionally, severe calcification at the IIA ostium may prevent smooth deployment. Furthermore, a key haemodynamic consideration is that the inner branch induces blood flow redistribution in the EIA and IIA. Thus, an extremely small true lumen (<10 mm) might pose a risk of limb occlusion due to the space occupied by the inner branch material. To rigorously address these issues, future research will involve a systematic *in vitro* mock circulation loop and animal *in vivo* studies. These studies will aim to clarify the optimal inner branch diameter to achieve ideal flow distribution and verify precise angle limits to prevent suture detachment or stent stenosis, thereby establishing standardised instructions for use for the future commercial IIBD.

### Study limitations

This study has two limitations. Firstly, its small sample size may introduce selection bias, which future multicentre studies will address to improve generalisability. Secondly, the results might have been more reliable had the CFD analysis of the PM-IIBD incorporated fluid structure interaction.

### Conclusions

This study, combining preliminary clinical and CFD analyses, demonstrated the efficacy and haemodynamic stability of novel PM-IIBD in CIA lesions. The PM-IIBD reduces spatial demands on the CIA, expanding the anatomic suitability for IBD technology, and could potentially be a feasible approach for IIA preservation in East Asians.

## Funding

This work was supported by the Natural Science Foundation of Beijing Municipality (7254314), 10.13039/501100001809National Natural Science Foundation of China (82170498), and the Beijing Science, Technology Planning Project (Z211100002921048) and Beijing Municipal Science and Technology Nova Interdisciplinary Program (20240484698).

## Ethical statement

The study was in accordance with the ethical standards of the Institutional Review Board of the Chinese PLA General Hospital (S2023-597-01) and Human Research Ethics Committee and with the 1964 Helsinki Declaration and its later amendments or comparable ethical standards.

## Conflicts of interest

The authors declare that they have no conflict of interest.
