# Supplementary material for: Clinical and Haemodynamic Evaluation of a Novel Physician Modified Inner Branch Iliac Branch Device for East Asians
Source: EJVES Vasc Forum. 2026 Jan 30;65:92–9. doi: 10.1016/j.ejvsvf.2026.01.005 (PMC13014961; doi:10.1016/j.ejvsvf.2026.01.005)
Supplement: Multimedia component 1 [file mmc1.pdf]

Supplementary Table S1. Computational Fluid Dynamics (CFD) parameter changes before and after PM-IIBD implantation in regions 1–4.

Parameter	Before PM-IIBD implantation	After PM-IIBD implantation	*p* value
**Region 1**			
Mean TAWSS	0.58 ± 0.19	0.68 ± 0.25	.33
Mean RRT	3.67 ± 1.94	2.81 ± 2.67	.49
Mean OSI	0.06 ± 0.02	0.05 ± 0.02	.14
Max TAWSS	12.51 ± 4.85	14.87 ± 5.68	.37
Max RRT	729.23 ± 788.63	293.91 ± 163.58	.56
Max OSI	0.44 ± 0.04	0.43 ± 0.02	.88
Percentage TAWSS > 4	0.59 ± 1.45	0.81 ± 0.89	.97
Percentage RRT > 20	2.62 ± 2.22	1.79 ± 3.18	.57
Percentage OSI > 0.35	0.22 ± 0.14	0.11 ± 0.07	.036
**Region 2**			
Mean TAWSS	0.48 ± 0.31	0.71 ± 0.22	.13
Mean RRT	4.38 ± 2.98	2.65 ± 1.7	.19
Mean OSI	0.07 ± 0.03	0.05 ± 0.02	.05
Max TAWSS	9.82 ± 6.03	13.81 ± 6.47	.15
Max RRT	420.48 ± 371.64	230.56 ± 139.17	.11
Max OSI	0.43 ± 0.04	0.42 ± 0.02	.49
Percentage TAWSS > 4	0.81 ± 2.34	1.18 ± 1.31	.25
Percentage RRT > 20	3.62 ± 3.61	1.52 ± 1.74	.16
Percentage OSI > 0.35	0.26 ± 0.21	0.14 ± 0.13	.10
**Region 3**			
Mean TAWSS	0.40 ± 0.18	0.72 ± 0.29	.015

Mean RRT	4.54 ± 4.03	2.74 ± 1.67	.24
Mean OSI	0.08 ± 0.03	0.06 ± 0.02	.046
Max TAWSS	4.47 ± 4.16	8.44 ± 6.52	<.0001
Max RRT	420.48 ± 371.64	222.23 ± 134.28	.094
Max OSI	0.42 ± 0.04	0.42 ± 0.03	.70
Percentage TAWSS > 4	0.17 ± 0.26	0.34 ± 1.34	.23
Percentage RRT > 20	4.97 ± 5.46	1.63 ± 1.76	.11
Percentage OSI > 0.35	0.3 ± 0.25	0.15 ± 0.11	.077
**Region 4**			
Mean TAWSS	0.40 ± 0.23	0.68 ± 0.22	.006
Mean RRT	5.52 ± 4.12	2.90 ± 1.93	.17
Mean OSI	0.07 ± 0.03	0.06 ± 0.02	.15
Max TAWSS	4.27 ± 4.78	7.50 ± 3.69	.22
Max RRT	370.84 ± 368.94	198.02 ± 125.5	.11
Max OSI	0.41 ± 0.05	0.42 ± 0.02	.64
Percentage TAWSS > 4	0.11 ± 0.39	0.20 ± 1.44	.002
Percentage RRT > 20	4.73 ± 5.94	1.85 ± 2.09	.57
Percentage OSI > 0.35	0.3 ± 0.23	0.14 ± 0.1	.098

Data are provided as mean ± standard deviation. CFD = Computational Fluid Dynamics; Max = maximum; PM-IIBD = physician modified inner branch iliac branch device; Region 1 = entire CIA; Region 2 = entire PM-IIBD; Region 3 = PM-IIBD main stent; Region 4 = PM-IIBD inner branch; TAWSS = time averaged wall shear stress, RRT = relative residence time, OSI = oscillatory shear index.
